# On the Value of Ergot in the Treatment of Pneumonia

**Published:** 1880-03

**Authors:** William Meacher

**Affiliations:** Portage, Wis.


### ARTICLE V.

### ON THE VALUE OF ERGOT IN THE TREATMENT OF PNEUMONIA.

By WILLIAM MEACHER, M.D., Portage, Wis.

About a year ago Dr. O. D. Colman, of Pardeeville, Wis., told me that he had used ergot in pneumonia with very gratifying results. He said that about six months before that, it occurred to him to try ergot in pneumonia. He had then used it in five or six cases, and in every case it seemed to cut the disease short by checking the extravasation of blood, so that there was no consolidation, or hepatization. I was greatly impressed with the idea at the time, and urged Dr. Colman to publish an article on the subject, but he neglected to do so. Since then the subject has been mentioned in several of the medical journals. But I cannot forbear giving my own experience with ergot in inflammation of the lungs. I have treated five or six cases, and Dr. Colman tells me he has treated fifteen or twenty with it. A history of my last case will serve to illustrate my experience. I copy from my case-book :

Was called January 13, 1880, to see Charley S., aged fifteen years ; found he had been attacked the day before with headache and chills ; he had high fever, severe pain in right side of chest, and cough ; pulse 120, temperature 103° ; prescribed Squibb's fl. ext. of ergot, five drops every three hours, and Dover's powders to relieve pain.

January 14th.—Less pain to-day, but high fever ; pulse 110, temperature 104° ; a little rusty expectoration, which commenced last night ; nearly the whole of the right lung seems to be inflamed ; the respiratory murmur is *harsh* over most of the lung. Continued treatment.

January 15th.—Pulse 100, temperature 103° ; less thirst and less pain to-day ; rusty expectoration almost stopped ; not much cough. Treatment the same.

January 16th.—To-day there was an attack of fever which seems to be of a malarial character. I found him with his pulse 110, temperature $104\frac{3}{4}$°. The lung no worse, apparently, and the rusty expectoration stopped. Prescribed quinia.

January 17th.—Fever came on again to-day at the same time as yesterday, showing it to be decidedly of a malarial character. Some mucopurulent expectoration ; respiratory murmur heard all over the lung, with some coarse râles ; pulse 100, temperature 103°. Continued quinia, but stopped the ergot.

January 18th.—To-day the temperature only reached 102°, pulse 90; very little cough, or trouble of any kind with the lung.

January 19th.—Patient got up to-day, and I did not see him again.

Now, here was a case that at the outset had all the symptoms of a bad case of inflammation of the lungs, but it seemed to be cut short by the ergot. There was very little extravasation of blood, and convalescence was very rapid, notwithstanding the malarial complication. Had it not been for the ergot in this case, I am satisfied that there would have been a good deal of extravasation and consequent hepatization, which most likely would have been serious and very tedious. Rapid convalescence has been the rule in the cases which I have treated with this drug. This has also been Dr. Colman's experience. Of course if I did not see the patient until hepatization had taken place, I should not think it worth while, then, to administer ergot, because the mischief, it is so potent to prevent, would already be done, unless extravasation was apparently still going on in some part of the lungs.

Although we do not know much about the cause of pneumonia, we know very well what the pathological condition is, and we know that pneumonia (I am speaking of it as it commonly occurs and wish to be so understood throughout this article), is serious or not, generally, in proportion to the amount of extravasation and consolidation. In pneumonia *something* affects the capillary vessels so that the blood leaks through their walls; this is how the mischief is done. Now, from what we know of the physiological action of ergot, its action on the unstriped muscular fiber and consequently on the walls of the capillaries, it is just the agent we should look to for the purpose of preventing this leakage.

I have no doubt that ergot will prove to be a most powerful remedy in the treatment of inflammation of the lungs, and enable the physician to render his patients, with this disease, real benefit by assisting nature, instead of hindering and embarrassing her in her efforts, as has been done to such a fearful extent in the past, by such drugs as veratrum viride, and other depressing agents.

